# Risk Factors and Neurologic Outcomes Associated With Resuscitation in the Pediatric Intensive Care Unit

**DOI:** 10.3389/fped.2022.834746

**Published:** 2022-04-04

**Authors:** En-Pei Lee, Oi-Wa Chan, Jainn-Jim Lin, Shao-Hsuan Hsia, Han-Ping Wu

**Affiliations:** ^1^Division of Pediatric Critical Care Medicine, and Pediatric Sepsis Study Group, Department of Pediatrics, Chang Gung Memorial Hospital at Linko, Taoyuan, Taiwan; ^2^College of Medicine, Chang Gung University, Taoyuan, Taiwan; ^3^Department of Pediatric Emergency Medicine, China Medical University Children Hospital, Taichung, Taiwan; ^4^Department of Medicine, School of Medicine, China Medical University, Taichung, Taiwan

**Keywords:** resuscitation, mortality, pediatric intensive care unit, cardiac arrest, neurologic outcome

## Abstract

In the pediatric intensive care unit (PICU), cardiac arrest (CA) is rare but results in high rates of morbidity and mortality. A retrospective chart review of 223 patients who suffered from in-PICU CA was analyzed from January 2017 to December 2020. Outcomes at discharge were evaluated using pediatric cerebral performance category (PCPC). Return of spontaneous circulation was attained by 167 (74.8%) patients. In total, only 58 (25%) patients survived to hospital discharge, and 49 (21.9%) of the cohort had good neurologic outcomes. Based on multivariate logistic regression analysis, vasoactive–inotropic drug usage before CA, previous PCPC scale >2, underlying hemato-oncologic disease, and total time of CPR were risk factors associated with poor outcomes. Furthermore, we determined the cutoff value of duration of CPR in predicting poor neurologic outcomes and in-hospital mortality in patients caused by in-PICU CA as 17 and 23.5 min respectively.

## Introduction

Cardiac arrest (CA) is uncommon in children, and the epidemiology of pediatric CA is different from adults. CA is a critical cause of death in children in the hospital especially in the pediatric intensive care unit (PICU). CA is reported in 2–6% of children in the PICU, which is much higher than out-of-hospital arrest (about 8 to 20 annual cases per 100,000 pediatric population) (1–3). Previous studies reported that return of spontaneous circulation (ROSC) after cardiopulmonary resuscitation (CPR) for in-hospital CA (IHCA) is about half of these patients, and ~30% survived to hospital discharge (1, 4, 5).

Previous studies had analyzed the cause and prognostic factors associated with outcomes after IHCA, which included the initial rhythm, duration of CA and CPR, the underlying disease, and where the event attacked (6). Patients in PICU are more likely to develop CA because they are more critically ill. Information on factors associated with prognosis of in-PICU CA can promote improvement in PICU care, which means improving survival with good neurologic outcomes. Analyzing the epidemiological variables and risk/prognostic factors of in-PICU CA is of great importance in developing the better therapeutic strategy and deciding appropriate preventive measures. The aim of the study was to analyze the clinical characteristics and prognostic factors associated with mortality and neurologic outcomes of in-PICU CA.

## Materials and Methods

### Patient Population

The study was conducted in the PICU of Chang Gung Hospital in Taiwan. Chang Gung Hospital is a tertiary medical center that received patients transferred from regional hospitals. These data were retrospectively analyzed from January 2017 to December 2020. All records of children aged from 1 month to 18 years who suffered in-PICU CA were included. As defined by the American Heart Association and the American College of Cardiology, CA is the sudden cessation of cardiac activity with clinical presentation, such as unresponsiveness to stimuli, apnea, or bradycardia with heart rate <60 bpm with poor perfusion that required external cardiac compressions and ventilation. The need for informed consent was waived by the Chang Gung Medical Foundation Institutional Review Board. This study was approved by the Ethics Committee of Chang Gung Memorial Hospital (approval no. 202000797B0).

Clinical data include age, birth weight, sex, previous history of CA and neurologic status based on the pediatric cerebral performance category (PCPC), underlying diseases, previous treatment, initial ECG rhythm, time to initiation of CPR, and total time of CPR.

Outcomes including return of spontaneous circulation (ROSC) and PCPC score were collected and analyzed. ROSC was defined as restoration of perfusion and heart rhythm in the absence of external chest compressions for over 20 min. The PCPC scores, ranging from 1 (normal) to 6 (brain dead), were validated to quantify a child's cognitive function after a critical illness or an injury. The investigators judged the PCPC score by reviewing the discharge summaries and outpatient records with the consensus of the pediatric neurologist. Categories 1 to 2 were viewed as good neurological outcomes.

The main outcomes for this study were mortality and neurologic outcomes at hospital discharge.

### Statistical Analysis

Univariate analyses were performed using the chi-square test, Fisher's exact test, or Mann–Whitney U-test, as appropriate. Multivariate logistic regression analysis with stepwise selection was conducted to identify independent predictors of main outcomes (mortality and neurologic outcomes). Finally, receiver operating characteristic (ROC) curve analysis was used to define the optimal cutoff values for the clinical parameters that may contribute to the main outcomes. A *p*-value < 0.05 was considered to be statistically significant. The IBM SPSS Statistics software (version 20.0; SPSS Statistics for Windows, Armonk, NY, USA) was used for all statistical calculations.

## Results

### Patient Demographics

**Figure 1:**
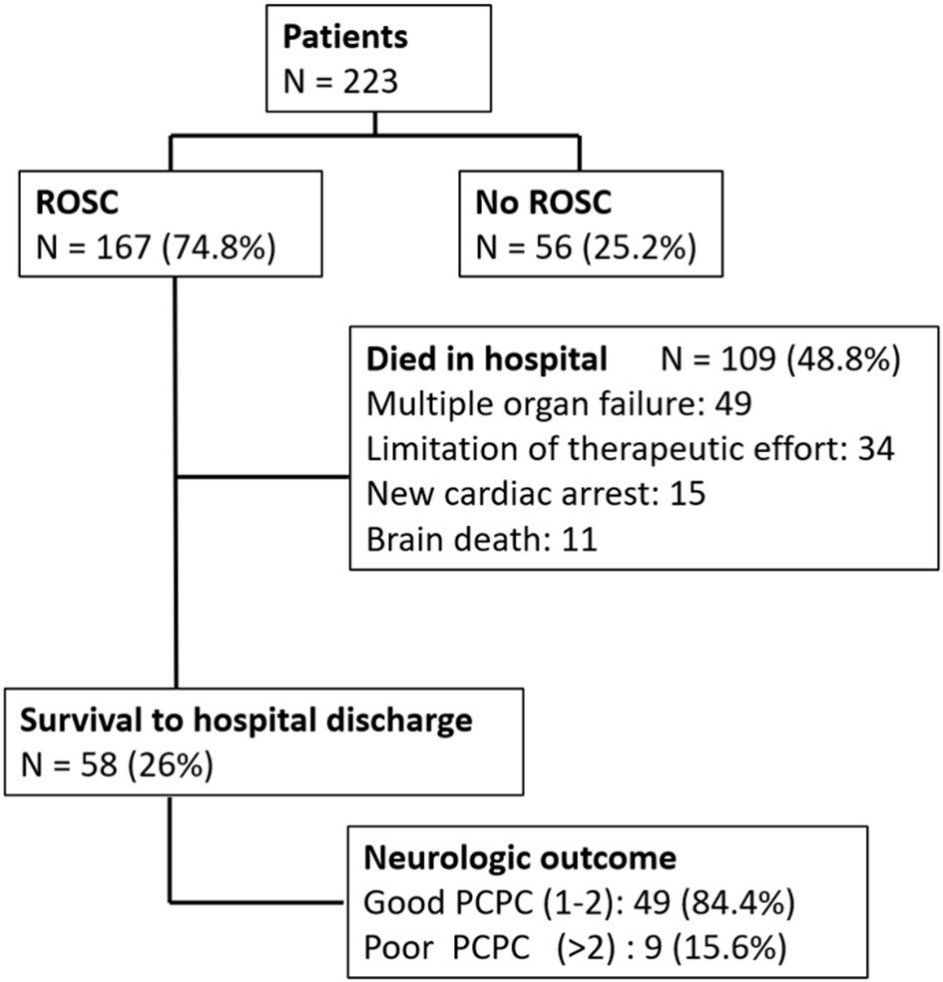
Algorithm of in-PICU CA. ROSC, return of spontaneous circulation; PCPC, pediatric cerebral performance category.

In this retrospective observational cohort study, we enrolled patients aged from 1 month to 18 years who suffered in-PICU CA between January 2017 and December 2020. Our PICU is a tertiary intensive care unit with 29 beds for patients aged from 1 month to 18 years. During the study period, there were 2,540 patients admitted in our PICU and a total of 223 (8.7%) in-PICU CA cases were retrieved. The return of spontaneous circulation was attained by 167 (74.8%) patients. In total, only 58 (25%) patients survived to hospital discharge, and 49 (84.4%) of the survivors had a good neurologic outcomes (Figure 1).

### Clinical State Before Cardiac Arrest and Characteristics of Arrest and Resuscitation

Table 1 reports the patients' neurologic outcomes and mortality rate of different characteristics. Most patients are aged from 1 to 12 months old (65.5% of the cohort). In all, 79.8% of these patients had an underlying disease. Patients with hemato-oncology disease had higher mortality, and patients with respiratory and neurological disease presented lower mortality.

**Table 1:** Clinical characteristics and risk factors associated with prognosis of in-PICU CA.

**Variables**	**All**	**Mortality (%)**	**Good**	**Poor**	***P-value***
**Patient number**	223	73.9	49 (21.9)	174 (78.1)	
**Age (n, %)**					<0.001
1 m – 12 months	146 (65.5)	72.6	37 (75.5)	109 (62.6)	
1 – 8 years	38 (17)	71	7 (14.3)	31 (17.8)	
> 8 years	39 (17.5)	82.1	5 (10.2)	34 (19.5)	
**Gender (n, %)**					0.38
Male	117 (52.4)	76.1	23 (46.9)	94 (54)	
Female	106 (47.6)	71.6	26 (53.1)	80 (46)	
**Underlying diseases (n, %)**					0.24
No	45 (20.2)	82.2	7 (14.3)	38 (21.8)	
Yes	178 (79.8)	71.9	42 (85.7)	136 (78.2)	
Prematurity	97 (43.5)	72.1	25 (51)	72 (41.4)	0.22
Hemato-oncology	25 (11.2)	92	2 (4.1)	23 (13.2)	0.04
Heart Disease	18 (8.1)	88.8	2 (4.1)	16 (9.2)	0.24
Neurological	13 (5.8)	38.4	3 (6.1)	10 (5.7)	0.92
Malformation	13 (5.8)	61.5	4 (8.2)	9 (5.2)	0.43
Respiratory	8 (3.6)	37.5	5 (10.2)	3 (1.7)	0.004
Digestive	3 (1.3)	66.6	1 (2)	2 (1.1)	0.63
Renal	1 (0.4)	100	0 (0)	1 (0.6)	0.59
**Previous PCPC scale (n, %)**					0.04
1 , 2	193 (86.5)	72.7	49 (100)	144 (82.8)	
> 2	30 (13.4)	81.3	0	30 (17.2)	
**Previous treatment (n, %)**					
**Mechanical ventilation**					0.47
Yes	168 (75.3)	75	35 (71.4)	133 (76.4)	
No	55 (24.7)	70.9	14 (28.6)	41 (23.6)	
**Vasoactive-inotropic drugs**					<0.001
Yes	134 (60.1)	85.8	16 (32.7)	118 (67.8)	
No	89 (39.9)	56.1	33 (67.3)	56 (32.2)	
**Bicarbonate**					<0.001
Yes	141 (63.2)	85.1	19 (38.8)	122 (70.1)	
No	82 (36.8)	54.8	30 (61.2)	52 (29.9)	
**Etiology of arrest (n, %)**					
Respiratory	85 (38.1)	55.3	31 (63.3)	54 (31)	<0.001
Cardiac	66 (29.5)	81.8	12 (24.5)	54 (31)	0.37
Sepsis	35 (15.7)	97.1	1 (2)	34 (19.5)	0.002
Neurological disease	18 (8.1)	77.7	2 (4.1)	16 (9.2)	0.24
Trauma	5 (2.2)	100	0 (0)	5 (2.9)	0.23
Others	14 (6.3)	78.5	3 (6.1)	11 (6.3)	1
**Time to initiation of CPR (n, %)**					<0.001
<1 min	202 (90.5)	73.7	45 (91.8)	157 (90.2)	
1-4 min	20 (8.9)	75	4 (8.2)	16 (9.1)	
4 - 10 min	0	0	0	0	
10−20 min	1 (0.5)	100	0	1 (0.7)	
**First cardiac rhythm (n, %)**					<0.001
Bradycardia	154 (69)	71.4	39 (79.6)	115 (66.1)	
Asystole	42 (18.8)	78.5	6 (12.2)	36 (20.7)	
Pulseless electrical activity	14 (6.4)	85.7	1 (2)	13 (7.5)	
Ventricular fibrillation or pulseless ventricular tachycardia	13 (5.8)	76.9	3 (6.1)	10 (5.7)	
**Total time of CPR (n, %)**					<0.001
<5 min	83 (37.2)	58.5	29 (59.2)	54 (31)	
6 – 10 min	39 (17.5)	73.1	7 (14.3)	32 (18.3)	
11 – 20 min	33 (14.8)	75	8 (16.3)	25 (14.4)	
21 – 30 min	17 (7.6)	76.5	4 (8.2)	13 (7.5)	
> 30 min	51 (22.9)	98	1 (2)	50 (28.7)	
ROSC	167 (74.8)	65.2	49	118	
**In-hospital mortality**	165 (73.9)		0	165 (94.8)	

* : p <0.05 statistic significant; p value was compared between groups of good and poor neurologic outcomes. PICU, pediatric intensive care unit; CA, cardiac arrest; PCPC, pediatric cerebral performance category; CPR, cardiopulmonary resuscitation; ROSC, return of spontaneous circulation.

One hundred ninety-three patients (86.5%) have good neurologic status (PCPC score of 1 or 2) prior to CA. After the CA event, 74.6% of the 193 patients developed to poor neurologic outcomes.

As for previous treatment, the patients who received vasoactive–inotropic and bicarbonate had significantly higher mortality rate than those who did not receive such drugs.

The two main etiology of CA were respiratory and cardiac disease. The rate of poor outcome was significantly higher in patients caused by sepsis.

In 90.5% of the cohort, CPR was initiated within 1 min after CA developed. Bradycardia (69%) was the most common cardiac rhythm, and only 13 patients (5.8%) presented with shockable rhythm.

CPR < 5 min accounted for the most proportion, and the longer CPR duration is associated with higher mortality.

### Association Between Clinical Risk Factors and Poor Outcomes

Multivariate logistic regression analysis was performed to investigate the impact of multiple risk factors in predicting poor neurologic outcome at discharge and in-hospital mortality. In the final model, only vasoactive–inotropic drug usage before CA, total time of CPR, and previous PCPC scale >2 remained as the independent predictors of poor neurologic outcome at discharge (Table 2). As for the risk factors of in-hospital mortality, only vasoactive–inotropic drug usage before CA, total time of CPR, and underlying hemato-oncologic disease remained as the independent predictors (Table 2).

**Table 2:** Multivariate logistic regression analysis for poor neurologic outcomes and in-hospital mortality.

**Variables**	**Odds ratio**	**CI 95%**	***p***
**Poor neurologic outcomes**			
Vasoactive-inotropic drugs usage before CA	4.15	2.03-8.48	<0.001
Total time of CPR, per minute increase	1.06	1.03-1.09	0.001
Previous PCPC scale > 2	4.96	1.08-22.68	0.039
**In-hospital mortality**			
Vasoactive-inotropic drugs usage before CA	4.94	2.46-9.92	<0.001
Total time of CPR, per minute increase	1.07	1.04-1.11	<0.001
Underlying hemato-oncologic disease	4.8	1.01-22.8	0.048

*CA, cardiac arrest; CPR, cardiopulmonary resuscitation; PCPC, pediatric cerebral performance category; CI, confidence interval*.

**Figure 2:**
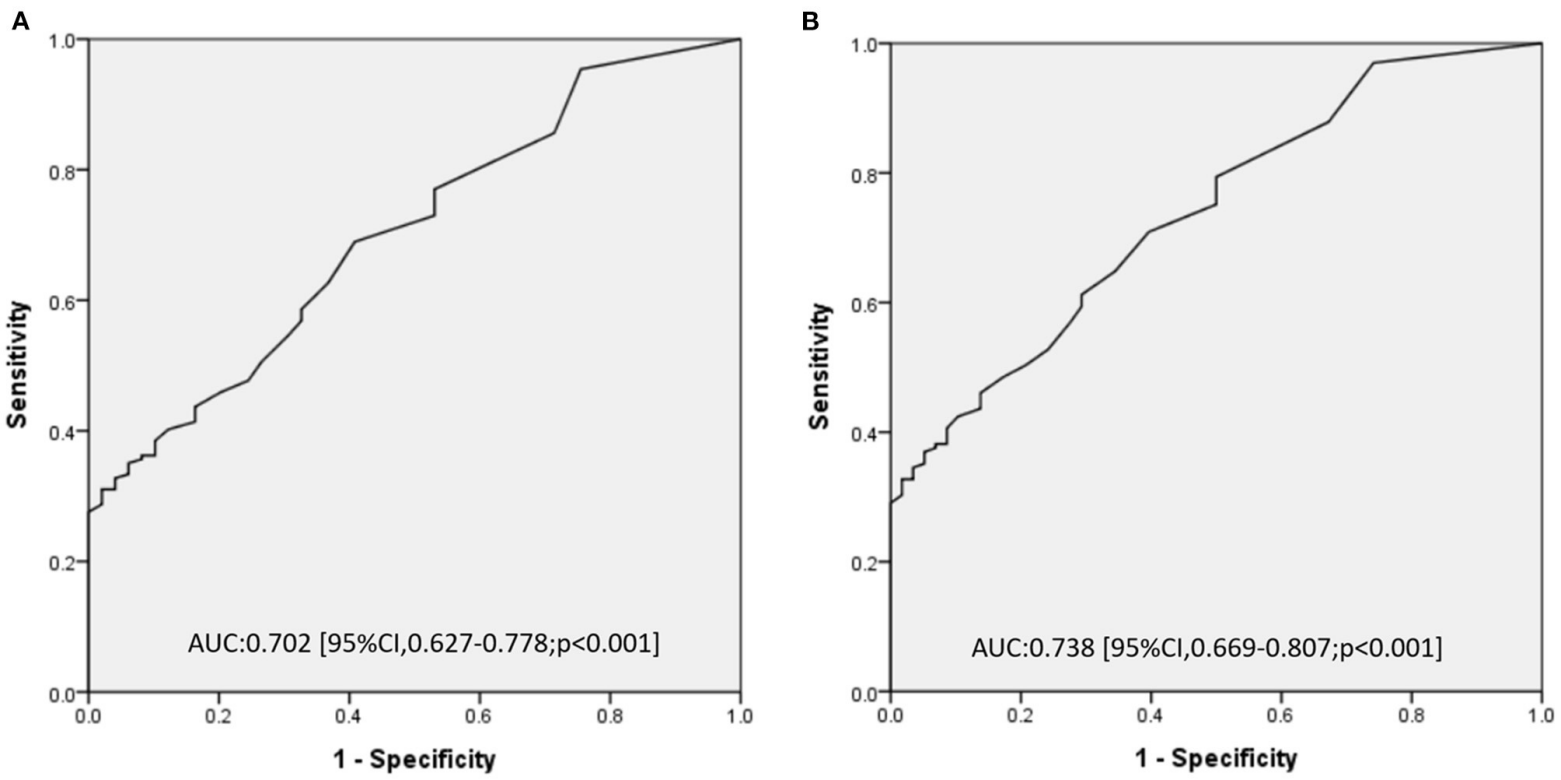
Receiving operating characteristic curve of total time of cardiopulmonary resuscitation (min) for predicting poor neurologic outcome **(A)** and in-hospital mortality **(B)**.

Figures 2A,B show the area under the ROC curve (AUC) of total time of CPR (min) for predicting poor neurologic outcome and in-hospital mortality. The AUC of total time of CPR (min) was 0.702 (95% CI, 0.627–0.778, *p* < 0.001) for poor neurologic outcome, and 0.738 (95% CI, 0.669–0.807, *p* < 0.001) for in-hospital mortality. The best cutoff values of total time of CPR (min) in predicting poor neurologic outcomes and in-hospital mortality are shown in Table 3. We identified total time of CPR (min) of 17 min for predicting poor neurologic outcome and 23.5 min for in-hospital mortality. Furthermore, the specificity was 99% for poor neurologic outcome after CPR for 29.5 min and was 99% for in-hospital mortality after CPR for 30.5 min.

**Table 3:** Best predictive power of the total time of CPR (min) for outcomes at discharge.

**Outcome**	**Total time of** **CPR (min)**	**Sensitivity**	**Specifcity**	**LR+**	**LR–**
Poor neurologic outcomes	17	0.385	0.898	3.77	0.68
	29.5	0.31	0.99	15.2	0.7
In-hospital mortality	23.5	0.37	0.95	7.15	0.66
	30.5	0.303	0.99	17.57	0.71

*CPR, cardiopulmonary resuscitation; LR+, positive likelihood ratio; LR-, negative likelihood ratio*.

## Discussion

The occurrence of pediatric CA is rare even in the relatively predictable settings, such as PICU. Previous studies have demonstrated the different rates of ROSC, in-hospital mortality, and neurologic outcome at discharge (Table 4) (7–9). The current study identified that in-PICU CA is associated with high mortality rate (73.9%), which is comparable with a previous study (1, 5, 7, 10). The purpose of the current study was to identify the independent risk factors associated with outcomes after in-PICU CA.

**Table 4:** Comparison different studies about in-PCIU CA.

**Author**	**Year**	**Type of study**	**Included patients**	**ROSC(%)**	**In-hospital mortality (%)**	**Good neurologic outcome at discharge (%)**
Meaney[7]	2006	Prospective, multicenter	411	48.9	78.6	14
Moreno[9]	2010	Prosepctive, one-center	132	53	52	16.6
Berens [10]	2011	Retrospective, one-center	257	56.8	68.9	19.8
Del Castillo[11]	2013	Prospective, multicenter	250	69.1	59.6	21.1
EP Lee	2020	Retrospective, one-center	233	74.8	73.9	21.9

*PICU, pediatric intensive care unit; CA: cardiac arrest; ROSC, return of spontaneous circulation*.

Sustained ROSC attained by 74.8% of patients and 26% of survivors were discharged with 21.9% of patients with good neurologic outcomes. The outcomes of in-PICU CA were better than the CA attacked in other areas, such as CA in the emergency department (6). Our previous study reported that only 4.6% of CA in the ED had a good neurologic outcome at discharge (11). Patients in the PICU received more intensive vital sign monitoring and readiness to recognize CA, then can immediately respond. ED has relative insufficient ability to monitor and stabilize patients competently. Training the medical team and improving the facility to identify and rescue CA quickly outside the PICU can improve the outcome (12, 13).

Previous studies had reported that several underlying diseases of patients before CA had impact on the outcome (7, 14, 15). The current study reported that respiratory patients have better neurologic outcome than those suffering from other disease. Most of the respiratory patients who suffered from CA were caused by obstruction of the airway, usually caused by sputum impaction of the airway or smaller size of the tracheal tube. Therefore, intensive care of the respiratory tract is very important for critically ill patients. Furthermore, patients with hemato-oncology disease had a significantly higher rate of poor neurologic outcome and in-hospital mortality, which was comparable with previous study (14). As for the etiology of CA, respiratory and cardiac diseases were most common, but the etiology of sepsis was the main cause of CA with increased poor neurologic outcome and higher in-hospital mortality in the current study. Sepsis is one of the main causes of admitting in PICU, so, it is important to build protocol for the early detection and resuscitation of sepsis.

Patients who received mechanical ventilation before CA did not suffer from higher morbidity and mortality. It may be because those in-PICU CA patients progress rapidly and did not need further respiratory exercise during CPR. Additionally, vasoactive–inotropic drug usage before CA was the independent risk factor associated with poor neurologic outcome and in-hospital mortality (16–19). Patients who need vasoactive–inotropic drugs indicating critical illness and hemodynamic disturbances, despite receiving intensive care, still had significantly higher morbidity and mortality.

Longer CPR duration after CA was a well-established risk factor associated with poor outcome, and previous studies identified CPR for more than 10 min as a prognostic risk factor (14, 15, 17, 18). Despite CPR for more than 10 min indicating poor prognosis, discontinuing resuscitation after CPR for 10 min seems to be too short. Deciding on the adequate duration of CPR before stopping resuscitation efforts in-PICU CA is a difficult subject, which needs the balancing of capability of the medical team, the family members' feelings, especially the probability of yielding extra morbidity. Therefore, we determined the cutoff value of the duration of CPR in predicting poor neurologic outcomes and in-hospital mortality in patients caused by in-PICU CA as 17 and 23.5 min, respectively, based on the ROC analysis. Furthermore, CPR for 29.5 and 30.5 min had about a 99% probability of poor neurologic outcome and in-hospital mortality, respectively. This information about CPR duration associated with poor prognosis can be used as a reference for some patients with extremely poor prognosis whether the resuscitation should be continued or stopped. Extracorporeal life support may be premediated for some patients with possible reversible disease, while they were not sustained ROSC (20–22). Further prospective studies are warranted to establish the guidelines.

1The current study has several limitations. First, this study was conducted at a single center with a retrospective design. Therefore, there is a risk of information bias, but we included a relative large patient number within the recent 3 years, which may be able to reduce the bias potentially. Second, the current study did not analyze the initial severity of illness at admission in the PICU, but other studies reported that there was no association between the initial severity of illness and the outcome of in-PICU CA (23). Third, rescue with extracorporeal membrane oxygenation (ECMO) during CPR was seldom performed in our PICU. Only two patients received ECMO CPR rescue caused by cardiac disease with a 50% mortality. ECMO CPR rescue may be considered for some patients with reversible disease but are insensitive to conventional CPR (18, 22). Further prospective studies are warranted to establish guidelines. Fourth, the quality of CPR performance is associated with prognosis that a high-quality of CPR is associated with better outcomes (24). A high-quality of CPR involves appropriate chest compression rate, proper chest compression depth, complete chest recoil, and minimization of compression interruptions, which were not analyzed in the current study.

## Conclusions

About one in five patients who suffered from in-PICU CA had good neurologic outcome, which had improved compared with previous studies. Several factors were associated with prognosis after in-PICU CA, such as vasoactive–inotropic drug usage before CA, previous PCPC scale >2, underlying hemato-oncologic disease, and total time of CPR. Furthermore, we determined the cutoff value of duration of CPR in predicting poor neurologic outcomes and in-hospital mortality in patients caused by in-PICU CA as 17 and 23.5 min, respectively.

## Data Availability Statement

The original contributions presented in the study are included in the article/supplementary material, further inquiries can be directed to the corresponding author.

## Ethics Statement

The studies involving human participants were reviewed and approved by the Institutional Review Board and Ethics Committee of Chang Gung Memorial Hospital. Written informed consent from the participants' legal guardian/next of kin was not required to participate in this study in accordance with the national legislation and the institutional requirements.

## Author Contributions

E-PL and H-PW conceived and designed the study. O-WC and J-JL participated in the data analysis. O-WC and S-HH gathered the data. E-PL drafted the manuscript. H-PW designed, oversaw the study, interpreted the data, and revised the manuscript. All authors have read and approved the final manuscript for publication.

## Conflict of Interest

The authors declare that the research was conducted in the absence of any commercial or financial relationships that could be construed as a potential conflict of interest.

## Publisher's Note

All claims expressed in this article are solely those of the authors and do not necessarily represent those of their affiliated organizations, or those of the publisher, the editors and the reviewers. Any product that may be evaluated in this article, or claim that may be made by its manufacturer, is not guaranteed or endorsed by the publisher.
